# Assessment of serum vitamin D levels in surgical adolescent idiopathic scoliosis patients

**DOI:** 10.1186/s12887-020-02114-9

**Published:** 2020-05-11

**Authors:** Abdulmonem Alsiddiky, Rheema Alfadhil, Maram Al-aqel, Noura Ababtain, Norah Almajed, Khalid Bakarman, Waleed Awwad, Raheef Alatassi

**Affiliations:** 1 Research Chair of Spinal Deformities, Department of Orthopedics, King Saud University, College of Medicine, P.O. Box: 3643, Riyadh, 11481 Saudi Arabia; 2 Department of Orthopedic Surgery, Security Forces Hospital, Riyadh, Saudi Arabia; 3 Department of Orthopedic Surgery, McGill University Health Centre, Jewish General Hospital, Montreal, Canada

**Keywords:** Idiopathic scoliosis, Vitamin D, Cobb angle, BMD, Alkaline phosphatase

## Abstract

**Background:**

The mechanism behind idiopathic scoliosis and its progression is not fully understood. Vitamin D insufficiency is known to play a role in the progression and/or occurrence of a variety of bone diseases. In this study, we aimed to estimate the prevalence of vitamin D insufficiency among patients with adolescent idiopathic scoliosis. Additionally, we aimed to calculate the differences in serum vitamin D levels, Cobb angles, spinal bone mass densities, and serum alkaline phosphatase levels between the sexes in the sample and to assess the possibility of a correlation between any of these factors.

**Methods:**

Demographic details, vitamin D levels, Cobb angle, spinal bone mass density, and alkaline phosphatase were collected from the records of 67 patients who were eligible for corrective surgery. These values were compared to normal levels and between the sexes within the study.

**Results:**

Of the 67 patients, 54 (80.6%) were female. The mean serum vitamin D level was 37.86 ± 26 nmol/L, and levels below normal were found in 92.5% of the patients. Statistical analysis showed significant differences (*p* = 0.002) in serum alkaline phosphatase levels between the sexes. No correlation was found between vitamin D levels and the Cobb angles, spinal and bilateral femoral neck bone mass densities, and serum alkaline phosphatase levels.

**Conclusions:**

Most adolescent idiopathic scoliosis patients had insufficient serum vitamin D levels and also suffered from low bone mineral density at an early age.

## Background

Adolescent idiopathic scoliosis (AIS) is a major public health problem and, despite being relatively rare with a prevalence of 0.47–11.1% [1], it reduces the quality of life. AIS is a three-dimensional deformity where there is a lateral curvature of the spine. The degree of spinal curvature is evaluated by the Cobb angle. A Cobb angle of more than 10–15° is considered pathological [2–4]. AIS occurs mostly in adolescents between 10 and 20 years of age and is more prevalent among females [3–7]. In general, scoliosis is considered severe and may require surgical intervention when the Cobb angle exceeds 40° [8].

The etiology of AIS is still unknown, but multiple theories have been described in the literature [1, 9–11]. Genetic and non-genetic factors have been proposed to cause AIS [12]. Among non-genetic factors is bone mineral density (BMD), as bone quality plays an important role in the derangement of bony mechanical stability. Osteoporosis is known to lower the bone strength and the prevalence of AIS with osteoporosis is approximately 20–38% [13, 14].

Vitamin D plays an essential role in maintaining a healthy mineralized skeleton. It helps with calcium absorption, and patients with a deficiency of vitamin D can have difficulties in producing new bone and maintaining their bone strength [1, 12].

Recently, several studies have suggested that a decrease in BMD may be responsible for the onset and progression of AIS, and some studies have tried to link vitamin D to adolescent idiopathic scoliosis [15, 16]. Despite this progression, there is a lack of studies on the link between vitamin D and AIS, and further studies are necessary to provide pooled evidence for the validation of the association of vitamin D with this clinical entity. Furthermore, exploring the association of vitamin D with AIS might contribute to the understanding of the pathogenesis of spinal deformities in AIS. Vitamin D deficiency is extremely common among the Saudi population despite its sunny weather [17, 18]. This study aimed to establish the prevalence of vitamin D insufficiency among patients with AIS as well as gender differences in serum vitamin D levels, Cobb angles, BMD, and serum alkaline phosphatase (ALP) level. Further, correlation of vitamin D with Cobb angles, BMD, and serum ALP levels was evaluated. We hypothesized that subjects with AIS would show vitamin D levels below normal and that these levels would be correlated with Cobb angles, BMD, and serum ALP levels.

## Methods

This retrospective cohort study was conducted in the orthopedic clinic at our institution, from August 2015 to October 2017. Clearance for the ethical approval was obtained from the hospital’s ethical committee prior to collecting the data.

Sixty seven consecutive AIS patients, including 13 (19.4%) males and 54 (80.6%) females, met the following study criteria: diagnosis of AIS, aged between 10 and 25 years old, Cobb angle of 40° or more (i.e. requiring corrective surgery), had their serum vitamin D levels measured prior to corrective surgery and had postsurgical follow-up of at least 1 year. Exclusion criteria were having non-idiopathic scoliosis, caused by congenital or neuromuscular conditions, being younger than 10 years or older than 25 years, and having no clinical record of serum vitamin D levels.

Patient clinical and demographic data, including age, sex, serum vitamin D level, Cobb angle, spinal and bilateral femoral neck BMD, and serum ALP were collected from electronic medical records.

Serum vitamin D [25-hydroxyvitamin D (25(OH)D)] levels were measured by electrochemiluminescence immunoassay (Roche, USA). Cobb angle measurement was obtained by measuring the largest spinal curve, taken from the upper-end vertebra to the lower-end vertebra, on x-ray for all patients included in the study. BMD values were taken from the spine and both femoral necks by using a dual-energy x-ray absorptiometry (DEXA) scanner (Lunar, GE medical systems, UK), as it is the most accurate way to measure BMD. Serum ALP was measured using a biochemical auto-analyzer (Siemens Health Diagnostics, USA). According to the manufacturer, normal vitamin D levels are ≥75 nmol/L and insufficient levels are < 75 nmol/L. The normal serum ALP level considered by the manufacturer was 50–180 IU/L.

### Statistical analysis

Data were analyzed using SPSS 21.0 (IBM, Armonk, NY). Descriptive statistics were represented as frequencies and percentages. A one-sample t-test was used to compare the mean vitamin D levels in AIS patients to normal levels. Student’s t-test was used to measure the association between categorical variables and outcome variables. A *p*-value of < 0.05 and 95% confidence intervals were used to report the statistical significance and precision of estimates. Additionally, a nonparametric correlation test was used to determine the correlation between vitamin D and the other study variables (Cobb angle, BMD, and ALP).

## Results

The records of 67 patients were included in the study; 54 (80.6%) of them were female, with a mean age of 16 ± 3.9 years (range, 10–25 years). Sixty-two (92%) of the patients had low vitamin D levels, with a mean level of 37.86 + 28 nmol/L. Thirteen (19.4%) of the patients had low spinal BMD, which was reflected by the low values in their DEXA scans. Moreover, 8 patients (11.9%) and 7 patients (10.4%) had low right and left femoral neck BMDs, respectively. The mean spinal BMD was 0.78 ± 0.26 g/cm^2^, and the mean right and left femoral necks BMD was 0.79 ± 0.13. The mean Cobb angle was 57.62 ± 20.5°, with a maximum of 122°. The mean serum ALP value was 170.86 ± 96.24 IU/L (Table 1).

**Table 1:** Baseline demographics of the scoliosis cohort (*N* = 67)

Variables	Number (%)
**Males**	13 (19.4%)
**Females**	54 (80.6%)
**Vitamin D Insufficiency**	62 (92.5%)
**Low spinal BMD**	13 (19.4%)
**Low Right NOF BMD**	8 (11.9%)
**Low Left NOF BMD**	7 (10.4%)
**Elevated Serum ALP**	6 (9%)
**Age (years)**	16 ± 3
**Vitamin D (**nmol/L)	37.86 ± 28
**Mean Cobb Angle (°)**	57.62 ± 20.5
**Spinal BMD (gm/cm**^**2**^**)**	0.78 ± 0.26
**Left NOF BMD (gm/cm**^**2**^**)**	0.79 ± 0.13
**Right NOF BMD (gm/cm**^**2**^**)**	0.79 ± 0.13
**Serum ALP (IU/L)**	170.86 ± 96.24

*BMD* Bone mineral density, *ALP* Alkaline phosphatase, *NOF* Neck of femur

The one sample t-test showed that there was a significant decrease in serum vitamin D levels of the patients with AIS compared to the normal serum vitamin D values of healthy individuals. The mean difference was 37.86 nmol/L (95% confidence interval [CI]: 31.6; 44.6, *p* < 0.001). The independent t-test showed no significant differences between the male and female groups regarding the vitamin D levels, Cobb angle, and BMD. The only significant variable was the serum ALP level, which had a mean difference of 120.06 (95% CI: 60.27; 179.84, *p* = 0.002) (Table 2). Spearman’s correlation testing revealed no significant correlation between vitamin D values, Cobb angle, BMD, and ALP levels (Table 3).

**Table 2:** Comparison of the study variables between the male and female groups (N = 67)

Variable	Male	Female	Mean difference	t	***p***-value	Confidence interval	
						Lower	Upper
**Vitamin D**	33.09 ± 16.4	39.36 ± 30.4	−6.27	−0.72	0.12	−23.74	11.19
**Cobb angle**	64.5 ± 24.56	56.12 ± 19.5	8.38	1.266	0.56	−4.86	21.62
**Spinal BMD**	0.81 ± 0.19	0.77 ± 0.29	0.04	0.38	0.49	−0.16	0.24
**Left NOF BMD**	0.82 ± 0.10	0.78 ± 0.13	0.04	0.913	0.34	−0.05	0.14
**Right NOF BMD**	0.78 ± 0.12	0.79 ± 0.13	−0.01	−0.19	0.52	−0.11	0.09
**ALP**	265.9 ± 125.53	145.84 ± 69.64	120.06	4.042	0.002	60.27	179.84

*BMD* Bone mineral density, *ALP* Alkaline phosphatase, *NOF* Neck of femur

**Table 3:** Correlation between vitamin D and other study variables

Variables	Correlation coefficient	***p***-value
**Cobb angle**	−0.16	0.29
**Spinal BMD**	0.11	0.47
**Right NOF BMD**	−0.13	0.36
**Left NOF BMD**	−0.14	0.39
**ALP**	−0.08	0.57

*BMD* Bone mineral density, *ALP* Alkaline phosphatase, *NOF* Neck of femur

## Discussion

We studied 67 post-operative AIS patients aged between 10 and 25 years, with the majority of them being females. In a study in Brazil, a similar female preponderance is documented. This suggests that females are more vulnerable to develop AIS [6, 7]. In the present study, vitamin D insufficiency was more common amongst our sample of AIS patients, which was consistent with previous studies which report that around 91% of patients show insufficient vitamin D levels [1, 2, 15, 16]. Serum vitamin D level was compared between male and female patients. We found that female subjects had higher levels than male subjects. However, the difference was not statistically significant. Significant differences were noted when comparing the vitamin D levels between patients with AIS and healthy subjects from international studies. The findings of the present study are in agreement with previous international studies [2, 15, 16]. A meta-analysis published in 2018 measured vitamin D levels among the Saudi adult population (between the ages of 18 and 80 years) in most regions, and they concluded that vitamin D deficiency was observed in 63.5% of the population [19]. To the best of our knowledge, no published studies have addressed the presence of vitamin D insufficiency/deficiency in the normal Saudi population of the same age group (10–25 years old).

The level at which the symptoms of Vitamin D deficiency become severe enough to make an impact on the skeleton are not known, neither in the normal population nor in AIS patients. This has led to a belief that a level of ≥50 nmol/L could be considered normal, so that insufficient vitamin D levels would be excluded and only normal vitamin D or vitamin D deficient categories would be necessary [19].

The values of Cobb’s angle and BMD were not statistically different between males and females in this current study. However, serum ALP was significantly different between male and female patients. Our study showed that serum ALP was found to be elevated in 6 subjects (9%); this is possibly due to the higher bone turnover rate exhibited by patients with AIS. Similar to the present study, elevated ALP levels have been reported in previous studies [20]. In a study by Shaheen et al., vitamin D-deficient subjects were grouped into mild, moderate, and severe groups according to their serum vitamin D levels. That study found that ALP levels were the highest in the moderate group and the lowest in the severe group [21].

Gozdzialska et al. [15] studied the relationship between ALP levels and sex hormones in pre- and postmenarcheal female subjects. The study concluded that the premenarcheal group had higher ALP values than the postmenarcheal group due to the inhibition of the receptor activator of nuclear factor kappa-Β ligand (RANKL) activity, which is known to have an antagonizing effect on osteoclasts within the bone [9–13, 22]. This could explain the significant difference in serum ALP levels found between males and females in this study. This difference could be explained by females generally having higher levels of circulating estrogen, especially during the age of puberty, which could reduce the action of osteoclasts by inhibiting the action of RANKL, leading to them having a lower bone turnover rate, which translates to lower and more controlled serum ALP levels.

A study conducted in one of the largest spinal units in Saudi Arabia [5], to identify the epidemiological patterns of scoliosis, noted that despite using the standard definition for scoliosis, which is a Cobb angle of 10° as the minimum lateral spinal angulation [6], most Cobb angles in Saudi patients fell within the surgical range (≥40°). The mean Cobb angle at presentation was 58°, which is similar to what was found in our study (57.62 ± 20.5°). We agree that scoliosis patients in Saudi Arabia requiring surgery are more prevalent, possibly due to the reduced awareness of back deformities within the community and the rather large portion of patients suffering from scoliosis as a complication of poliomyelitis.

The mean Cobb angle in the present study was 57.62 ± 20.5°, with a maximum angle of 122°. A study done by Lee et al. [8] in Hong Kong studied the extent of Cobb angle severity in relation to BMD status. The subjects in this study were grouped into moderate and severe groups according to their Cobb angles. This indicates that most surgical cases, such as those used in our sample, were considered severe cases in other countries in the far east. They stated that the axial and peripheral BMDs and bone mass content (BMC) in the moderate or severe AIS groups were significantly lower than in controls. They concluded that the higher the Cobb angle, the lower the volumetric and areal BMD and BMC. Despite the difference in geographical location, the results we found were rather similar.

Sadat-Ali et al. [23] compared scoliotic girls with healthy counterparts. BMD was found to be significantly lower in the scoliotic patients, and patients with a Cobb angle of > 30° had significantly lower bone mass compared to those with a Cobb angle of < 30°. Our study revealed that 13 (19.4%) of our subjects appeared to have low spinal BMD, which is in agreement with the findings of the aforementioned study [22]. Ishida et al. [13] reported that the lumbar spinal BMD of AIS patients was 0.9 g/cm^2^, with 51% being osteopenic and 14% being osteoporotic. They concluded that there was a significant correlation between the severity of scoliosis and BMD. The overall decrease in BMD in our study could be due to the increase in bone turnover rate observed in AIS patients. Cheng et al. also found a significant decrease in BMD in AIS subjects [22].

### Limitations

We are aware of several limitations of our study. First of all, the use of controls is designed to minimize the effects of variables other than the independent variable. It would increase the result reliability and would reflect a true image of the normal population. However, in this study, no gender, age, and ethnic-matched controls were ascertained. We understand that having controls may have a significant impact on the results and may change the results completely. Yet, in our study, we compared the means against average values published for the Saudi population. Second, this is a retrospective study, no causal relationship between low vitamin D levels and AIS can be established. Third, ALP levels measured in this study were not bone-specific ALP, which may have affected the results presented from that aspect. Fourth, the small sample size and the fact that this was a single-center study. As a result, we recommend performing prospective multicenter studies with larger series of patients to validate the association of vitamin D in the etiology of AIS. In addition, further studies are required to determine vitamin D levels before and after corrective surgery.

## Conclusion

This study revealed that most AIS patients have insufficient serum vitamin D levels. Furthermore, most AIS patients are also suffering from low BMD at an early age. The study variables were not significantly different between males and females (excluding serum ALP), which indicates that the severity of AIS is not different between sexes. Although low levels of vitamin D were associated with higher Cobb angles, statistical significance could not be reached. Furthermore, no correlation was found between vitamin D levels and measures of BMD or serum ALP.

## Data Availability

The datasets used and analyzed during the current study are available from the corresponding author on reasonable request.

## Abbreviations

AIS: Adolescent Idiopathic Scoliosis
BMD: Bone Mineral Density
ALP: Alkaline Phosphatase
RANKL: Receptor Activator of Nuclear Factor Kappa-Β Ligand
NOF: Neck of femur
